# Brightness/darkness induction and the genesis of a contour

**DOI:** 10.3389/fnhum.2014.00841

**Published:** 2014-10-20

**Authors:** Sergio Roncato

**Affiliations:** Dipartimento Psicologia Generale, Università PadovaPadova, Italy

**Keywords:** brightness perception, layered images, contour integration, contrast polarity, optical illusions

## Abstract

Visual contours often result from the integration or interpolation of fragmented edges. The strength of the completion increases when the edges share the same contrast polarity (CP). Here we demonstrate that the appearance in the perceptual field of this integrated unit, or contour of invariant CP, is concomitant with a vivid brightness alteration of the surfaces at its opposite sides. To observe this effect requires some stratagems because the formation in the visual field of a contour of invariant CP normally engenders the formation of a second contour and then the rise of two streams of induction signals that interfere in different ways. Particular configurations have been introduced that allow us to observe the induction effects of one contour taken in isolation. I documented these effects by phenomenological observations and psychophysical measurement of the brightness alteration in relation to luminance contrast. When the edges of the same CP complete to form a contour, the background of homogeneous luminance appears to dim at one side and to brighten at the opposite side (in accord with the CP). The strength of the phenomenon is proportional to the local luminance contrast. This effect weakens or nulls when the contour of the invariant CP separates surfaces filled with different gray shades. These conflicting results stimulate a deeper exploration of the induction phenomena and their role in the computation of brightness contrast. An alternative perspective is offered to account for some brightness illusions and their relation to the phenomenal transparency. The main assumption asserts that, when in the same region induction signals of opposite CP overlap, the filling-in is blocked unless the image is stratified into different layers, one for each signal of the same polarity. Phenomenological observations document this “solution” by the visual system.

## Introduction

The perceptual world is the product of complex building strategies able to organize fragments into structures or to reorganize a structure into a different one. Sometimes these processes originate from a small variation in figural or luminance cues.

**Figure 1:**
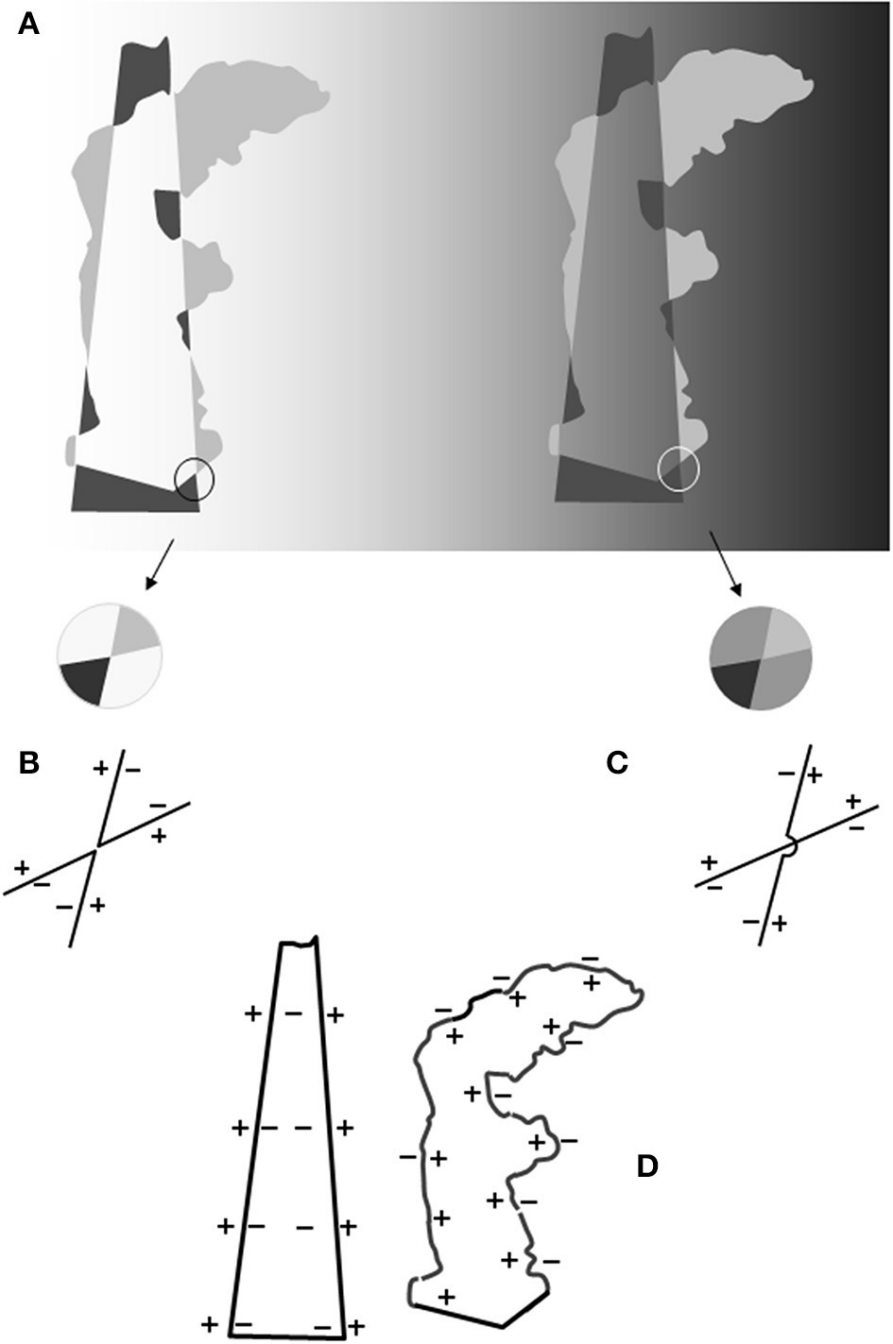
**(A)** Left side: irregular shapes against a lighter background. Right side: a replica of the pattern of irregular shapes drawn on the left side against a background of a mid-gray shade. Note that the shapes are lighter or darker than the background. The spatial and photometric relationships follow the building schema of the “argyle illusion” (Adelson, 1993) so as to elicit the impression of a percept organized into two layers: an opaque figure, i.e., the Greek statue known as the “Winged Victory,” and a translucent filter, i.e., a shadow cone obscuring part of the statue's silhouette. The two contours and their contrast polarities (CP) are symbolized in the outlines in **(D)**. Note that in the space embedded within the two profiles (invariant CP axes) positive induction signs converge in the cone and negative induction signs converge in the statue. **(B)** Enlargement of a portion of the leftmost pattern and the outline representation of the corners nearby with the “+” and “−” symbols indicating the lighter and darker sides of the edges (CP). A *double-reversing X-junction* (Adelson and Anandan, 1990) appears, so-called because the prolongation of a corner side meets an edge with the opposite CP. The CP persists, but along the corner perimeters. No edge merging is expected in this condition. The contours we perceive coincide with the perimeters of the irregular gray shapes. **(C)** Enlargement of the correspondent portion in **(B)** following the darkening of the background. A *double-preserving X-junction* is depicted, so-called because the prolongation of the corner edge encounters an edge of the same CP. We assume that in this condition the edges of two opposite corners perceptually merge as we have shown in the crossing schematics. The outline drawing symbolizes the result: the two acute angles of **(B)** are now reassembled as two intersecting linear edges. In other words, the transformation of a *double-reversing* to a *double-preserving X-junction* involves a figural reorganization such that two adjacent corners are perceived as two intersecting edges. The layered representation of the image is not predicted by Metelli's (1974) rules that apply in a context of *single-reversing X-junctions*. **(D)** The two contours of invariant CP resulting from the cohesive events described in **(C)**. Note that the two contours have an opposite CP. Within the shadowed surface the symbol “−” indicates a propagation of darkness signals (the shadow appears darker than the background). The inside border of the “winged victory” is traversed by “+” signals (the statue looks lighter than the background). When superimposed these contours will generate induction signals of the opposite sign within the same area.

A curious example is depicted in Figure 1 where a white to black gradient background makes the silhouette of a Greek statue to appear. In this figure the same set of irregular shapes is reproduced twice: on the left against a white background, on the right against a faded mid-gray. This change generates strong cohesive “forces” since the meaningless group of shapes depicted on the left transforms into a complex figural organization on the right. The silhouette of the “Winged Nike Victory of Samothrace” (standing on the grand staircase landing on the Louvre museum) is perceived against a dark gray background partially obscured by a shadow cone.

This organization is rather complex and results from contours and surfaces completion. Furthermore, it appears stratified in different layers, one for an opaque white figure and the other for a transparent dark filter (shadow). In the region where they overlap a luminance scission occur: the gray surface seems to illusorily split into a bright and a dark layer. It is a further demonstration that the brightness (1) illusions, transparency and tridimensional organization of the percept are interrelated percepts (Nakayama et al., 1990).

The “optical effects” of configurations such as the “winged victory,” i.e., light and dark shapes (inducers) alternating in shade and position at the opposite sides of an imaginary line, were first illustrated by the Bauhaus designer Joseph Albers. We have discovered the importance of these illusions in vision science thanks to the studies of Adelson who created a series of astonishing demonstrations. The “argyle” (Adelson, 1993) and “snake” (Adelson, 2000) illusions are two of the most vivid demonstrations of brightness alteration arising in the perceptual world. Observers asked to describe patterns of alternating light/dark and dark/light columns of inducers (“argyle illusion”) reported the impression that portions of the background appeared darker and covered by a light filter whereas other portions seemed lighter covered by dark filter. These reports indicate a decomposition of the image into two layers: one for a reflectance and the other for a luminance (translucent) pattern. Adelson argued that the altered brightness of the background was the outcome of the “process of discounting the overlying filter” to recover background surface reflectance.

Other researchers based their work on the key assumption that brightness alterations may originate in an image stratified into layers. Two main goals were pursued: (i) To unveil the computations at the origin of the decomposition into layers; (ii) To show the ways the scission can induce transformations in brightness.

The computations underlying this decomposition depend critically on the boundaries between the targets and their surrounds; according to Adelson and Anandan (1990) the visual cues used to identify the presence of non-uniform are patterns of four convergent surfaces or X-junctions. The cross point may coincide with a contrast polarity reversal in both the intersecting contours (double-reversing junction). In this case no transparency percept arises. The contrast polarity may reverse in one direction only (single-reverse junction) or persist in both directions. These patterns are consistent with a transparency interpretation: unidirectional (unique) in the former case, bidirectional in the latter.

Anderson and colleagues (Anderson, 1997; Singh and Anderson, 2002) demonstrated that the scission into layers requires a more complex computation, an algorithm by which to process the geometric continuity, the contrast magnitudes, and their sign. Singh and Huang (2003) presented an algorithm detecting X-junctions, their contrast polarity, the circuits of polarity preserving X junctions, and ending with a decomposition of the image into layers.

In a stratified image both the opaque and translucent layer may show an alteration in brightness (Anderson, 1997, 1999, 2003a,b; Kingdom et al., 1997; Anderson and Winawer, 2005; Tse, 2005). The induction effects are opposite in the two layers: if the transparent layer dims, it appears to overlie a brighter opaque image; if the upper layer lightens, a dimmer surface is seen beneath.

The claims that the brightness illusions such as the “snake” and “argyle” occur through interposition of a transparent layer have been confuted by several authors who demonstrated the persistence of the illusory phenomena despite the removal of the conditions for transparency perception (Logvinenko, 1999; Bressan, 2001; Logvinenko and Ross, 2005; Albert, 2006). Furthermore, a presence of a translucent layer may not entail a brightness alteration (Logvinenko, 2002). Albert (2007) proposed an algorithm for computing lightness that does not assign a direct role to layering in brightness alteration. Further criticism of the layered representation approach has been reviewed by Gilchrist (2006) and Kingdom (2011). More recently, the image decomposition mechanism has been challenged by Todorović and Zdravković (2014).

All these findings suggest an approach to Figure 1 illusions different from the “image decomposition and discounting mechanism.” There is no doubt that the right image appears as stratified in a translucent and a reflectance pattern but it is not a foregone conclusion that the layering is the cause of the brightness alterations. Nothing prevents us from thinking that the brightness illusions and the split into layers are contemporary events arising from the effects of a common basic phenomenon. Such a conjecture can be tested only through a deeper reconstruction of what occurs when edges are integrated to form a contour. When, as in Figure 1, the sides of the irregular shapes reassemble to appear as the “winged victory” the phenomena of brightness alteration are likely to be present and contribute to the layering effect. The main purpose of the present work is to document the interaction between the phenomena of contour integration and brightness induction. The properties of these integrated figural units have been explored only in part and new data will contribute to the understanding of the vivid illusory effects that have been documented by Adelson and successive researches.

### A contour preserving the contrast polarity

The straight illuminant border in Figure 1 (the shadow cone), like the filter borders in similar patterns, has been considered a source of essential information for the computations to generate a layered image. The crucial role should be played by the X-junctions, a pattern of four converging surfaces that convey the information necessary for the split into layers (see Figure 1 caption).

**Figure 2:**
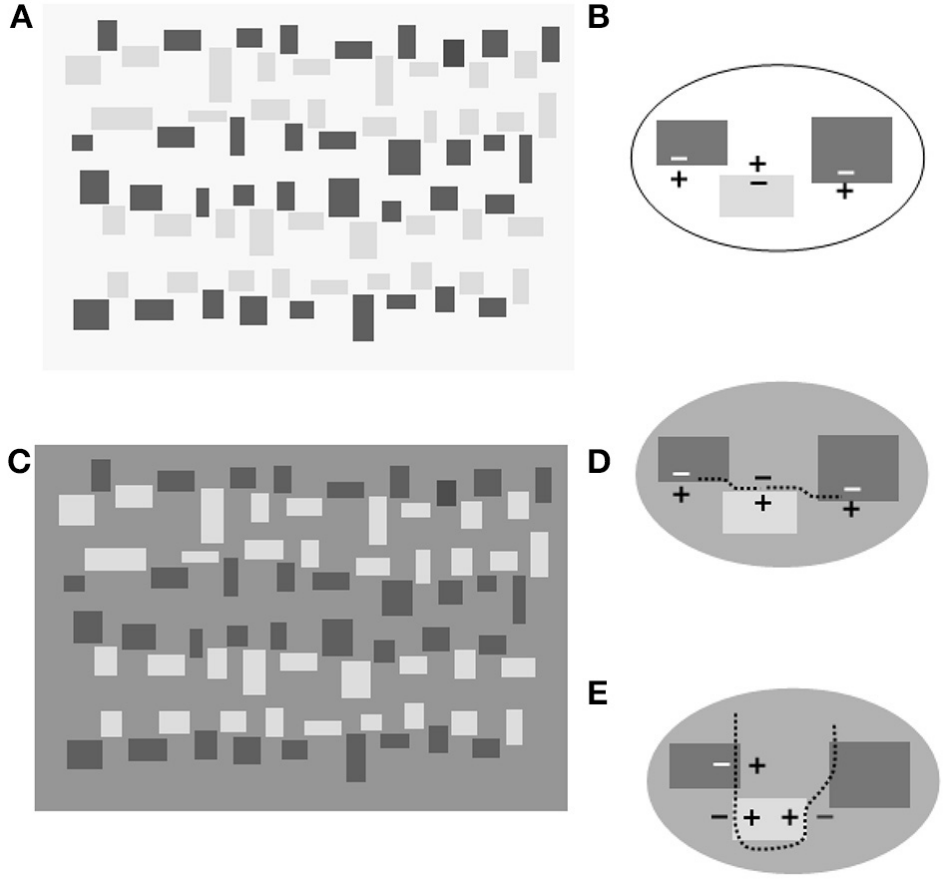
**(A)** Rectangles of different sizes arranged in irregular rows against a background of a lighter shade. **(B)** Enlargement of a portion of **(A)**: “+” and “−” indicates the lighter and darker sides, respectively. Note that the central edge inverts the contrast polarity (CP) with respect to the flanking edges. **(C)** The reproduction of **(A)** with the background luminance intermediate between the dark and light rectangles. The rectangle corners are not in contact so that no X or T-junction results. Despite the spatial gaps, an irregular contour seems to interpose between the light and the dark rectangles to form a border appearing like a dark veil overlying a surface divided into light and dark regions. **(D)** Enlargement of a portion of **(C)** illustrating the edges CP and the genesis of a contour (or *axis* of invariant CP). The edges are of the same CP so that they are expected to join. The doted curved lines symbolize the path of edge conjunctions. These local cohesive events sum to give rise to an overall effect that will be perceived as the border of a translucent surface. **(E)** Enlargement of a portion of **(C)** illustrating the edges and the genesis of a second contour of invariant CP. The doted curved lines symbolize the path of edge conjunctions. These local cohesive events sum to give rise to an overall effect that will be perceived as the border of the opaque surfaces.

But a simple configuration confirms the criticism of those who questioned this role. In Figure 2C a layered representation appears in violation of the geometrical constraint that has to be obeyed in order to evoke a phenomenal transparency. Small rectangles of a different size and gray shade are arranged in irregular rows. Each dark rectangle is spatially contiguous to a lighter one but separated at the corners by a gap of variable length. In Figure 2 the same configuration of light and dark gray rectangles is depicted twice: against a white background (Figure 2A) or against a mid-gray surface, or a surface of intermediate luminance between the shade of the dark and light rectangles (Figure 2C).

The variation in background luminance produces the same effects observed in Figure 1: the separated figural units organize into a stratified image in which opaque and translucent layers are visible. Dark and light bands horizontally traverse the whole configuration as transparent filters; their borders follow the irregular pathway separating the dark rectangles from the light ones.

Also the background appears to be divided into zones of different grayness, but, differently from the horizontal bands, which appear as opaque surfaces. When covered by a light veil they look darker; when covered by a dim veil they look brighter (see Figure 2 caption).

Therefore, in Figure 2C we see a replica of the “argyle illusion” effects, i.e., brightness alterations of a layered image (Adelson, 1993). Nevertheless, a question arises in regard to the relationships between the layering effect and the brightness alteration. The former has not a clear origin in Figure 2C because the necessary cues to decompose the image into layers are not drawn: both X and T-junctions are absent as well as occlusion cues. In light of this, it is hard to defend the causal role of the “image decomposition” for the rise of brightness illusion; it is reasonable to argue that layering is a derivative effect of a basic autonomous phenomenon of brightness/darkness induction.

Here I argue that the crucial event is not the formation of the X-junctions but rather the binding of fragmented edges into an illusory contour in which the contrast polarity (CP hereafter) is preserved.

This event is concomitant to the emission of brightness/darkness induction signals that alter the brightness in opposite directions at the two sides of the illusory contour.

This conjecture predicts, therefore, both contour completion and brightness induction effects.

Several experimental findings document a strengthening role of the CP in perceptual completion and grouping. Gabor units sharing the same CP are easier to perceptually group when distributed among other Gabor units (McIlhagga and Mullen, 1996; Field et al., 2000); real or illusory contours are easier to detect when of the same CP (Cavanagh and Leclerc, 1989; Spehar, 2000). Research on orientation misperception (Roncato and Casco, 2003, 2006; Van Lier and Csathó, 2006) demonstrated that this tendency manifests locally as illusory tilts at the edge extremities when they have the same contrast polarity and extend within a spatial range of 7–15°.

These geometric and photometric conditions are met in the corner regions of Figure 2C where the horizontal sides of the corners have the same CP and are separated by a narrow gap. Therefore, a contour binding phenomenon is expected to occur. The same event is predicted to arise in the perpendicular direction to bind the vertical sides of the corners.

The two completion phenomena are schematized in the enlargements of Figures 2D,E where dotted lines symbolize the two pathways of edge binding. These are the result of local phenomena that sum to give rise to an overall effect of an illusory contour passing through the whole surface extension.

The appearance of opposite induction effects at the two sides of an illusory contour are well known phenomena (Gerbino and Kanizsa, 1987; Petry and Meyer, 1987). Nevertheless, the brightness alterations in Figure 2 cannot be reduced to contrast enhancement and assimilation effects one may observe in the classic Kanizsa's edge (or line-end) induced illusory contours. The figural and photometric conditions in which the contours complete are different in the two cases. In particular it has to be stressed that in Figure 2 the edges binding occurs in two interlacing directions. The inducing effects following these completion phenomena interfere in a complex way.

In order to document the brightness alteration following a contour completion a simpler figural context has to be created so that the basic phenomena may be observed in isolation.

### Contour completion in Figure 2

**Figure 3:**
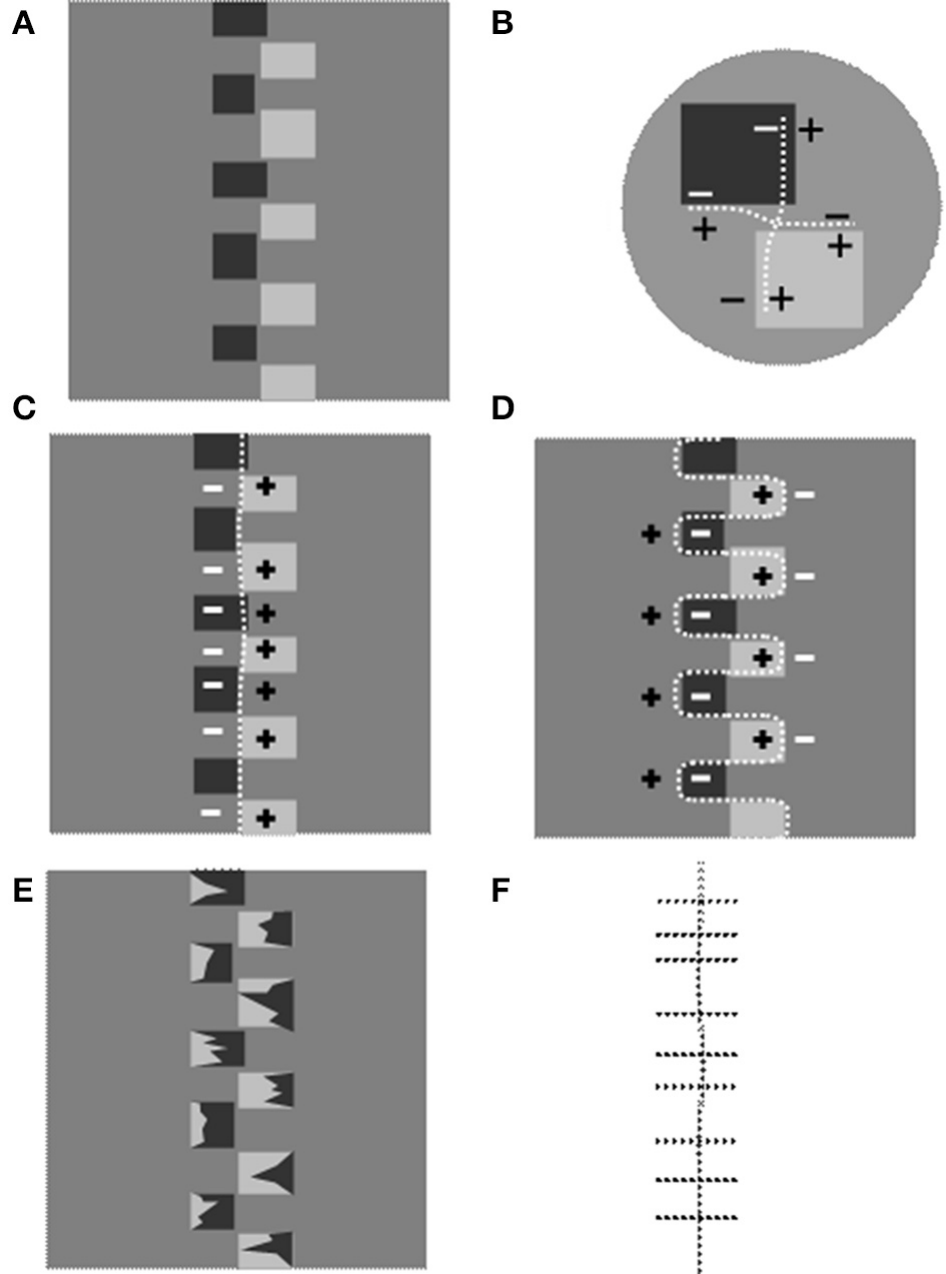
**The genesis of contour of invariant contrast polarity in Figure 2. (A)** A portion of Figure 2 vertically reoriented. **(B)** The two binding phenomena in the corner regions of Figure 2C. The dotted lines indicate the conjunction pathways. **(C)** The dotted line indicates one of the two axes (*axis* 1 hereafter) of invariant CP resulting from the chaining of the local binding effects in the corner regions. **(D)** A dotted line follows the outer perimeter of the rectangles to indicate the second *Axis* of invariant CP (*axis* 2 henceforth) resulting from the chaining of the local binding effects: the horizontally directed ones in **(B)**. **(E)** The rectangles in **(A)** are occluded, at their extremes, by irregular shapes of opposite contrast polarity. The reversal of the contrast sign inhibits the chaining process so that only the *axis* 1 survives. **(F)** The overall result of binding effects after the manipulation illustrated in **(D)**: the vertical *axis* 1 and horizontal binding effects that do not integrate to complete an illusory contour.

In Figure 3A a portion of Figure 2 is depicted vertically reoriented. In Figure 3B an enlargement of a corner region illustrates the contrast polarities at the edges and the two directions of the binding effects. A series of local bindings, as such, sum to give rise to two overall effects symbolized by dotted lines in Figures 3C,D, respectively. Note that the former follows a vertical pathway to bind the square sides vertically aligned (*axis* 1). The latter follows a waving pathway to circumvent the outer square perimeters (*axis* 2). There result, then, is the overall chaining effects that interpolate the local binding phenomena.

The hypothesis is that both the *axes* are sources of brightness induction effects. The appearance of this unit in the perceptual field is accompanied by a phenomenon of brightness/darkness induction: we perceive a dimmer gray shade on its negative side and a brighter gray shade on the opposite side.

In other words, we suppose these contours of invariant CP to behave as a source of brightness induction signals as the continuous edges prove to be when the so-called process of edge integration occurs (Grossberg and Mingolla, 1985; Rudd and Arrington, 2001; Rudd and Zemach, 2004).

Testing this hypothesis requires two steps.

First of all the conditions must be reproduced that allow us to observe the brightness induction of one *axis* acting in isolation. Second, we have to document what happens when two *axes* of invariant CP give origin to opposite polarity signals in the same region of perceptual space.

This overlapping of signals of opposite CP is illustrated in Figure 3. In Figure 3B the *axes* of invariant CP delimit quadrants in two of which “+” and “−” signals diffuse and overlap. Let's consider the left half of the background in Figure 3A. It is passed through by negative signals induced by *axis*1 (Figure 3C) and by positive signals from *axis* 2 (Figure 3D).

A simple stratagem allows us to disentangle the induction effects of *axis* 1 from the ones propagating from *axis* 2. This is illustrated in Figure 3E. Irregular shapes are superimposed on the outer extremes of the rectangles so that the inducers are split into two halves of approximatively the same area and opposite gray shade with respect to the intermediate gray surround (“twin-shade inducers” from now on). This variation leaves the CP along the *axis* 1 unaltered, whereas the pathway along the external perimeter of the shapes reverses the CP several times. Consequently the *axis* 2 cannot complete as Figure 3F illustrates.

The consequences on the brightness perception are not vivid in Figure 3E but if the same configuration is inserted in a repetitive pattern they clearly emerge.

**Figure 4:**
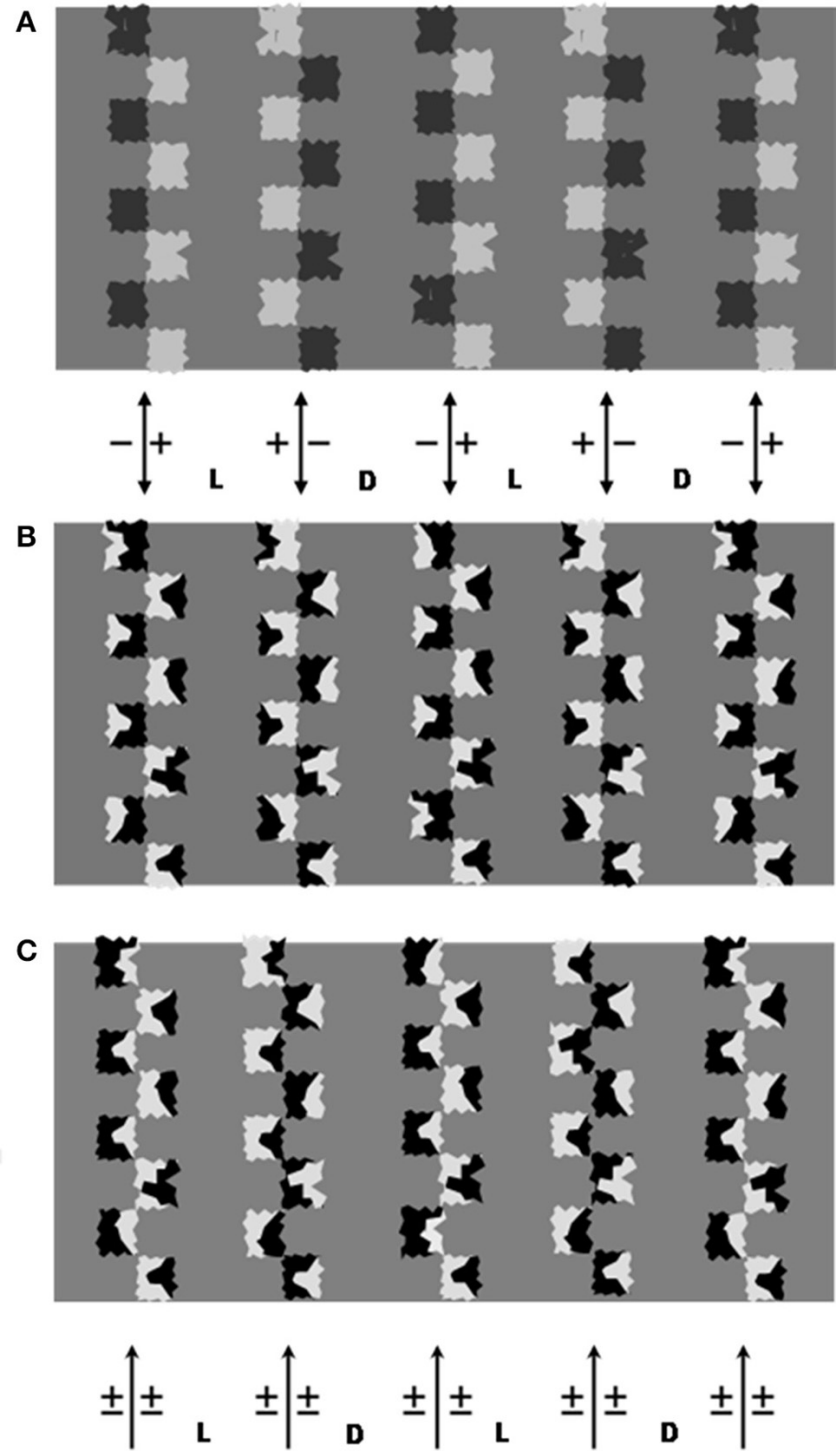
**The three figures have the same homogeneous gray background. (A)** Columns of jagged squares alternating in shade at the opposite side of a vertical dividing line. The building schema is the same as the Adelson's “argyle illusion.” Note the illusory alternation of dark and light gray regions. Opaque regions similar to piles of road signs alternate in dark and light gray shades. Vertical bands alternate as shadow or illuminated zones. **(B)** Columns of irregular shapes split into dark/light surfaces are used to generate vertical invariant CP axes. The basic drawing is identical to **(A)**, irregular shapes have been superimposed on the squares so that the CP reverses along their outer contour. The vertical axes are indicated by the bidirectional arrows. The perception of alternating dark and bright bands on the background is illusory, since they have the same luminance. Note that the background appears to dim when embedded between +/− −/+ axes and to brighten when embedded between and −/+ +/− axes. Labels D and L indicate the regions between +/− −/+ and −/+ +/− axes, respectively, and are regions that appeared as dimmed or lightened, respectively, in the test condition “divided background” (see text). **(C)** The same as **(A)** but with inducers on the left of the column mirror-imaged. Whatever pathway is followed, the CP inverts periodically. The arrows below point to the axes of variable CP (±). Labels D and L indicate the corresponding regions that were progressively darkened or lightened when the figure served as comparison stimulus.

In Figure 4 different configurations of inducers have been created so that to allow the comparison of the effects generated by or two interlaced contours of invariant CP. Inducers with a jagged contours were drawn in order to put a further obstacle to the rise of the phenomenal transparency. The three configurations have been depicted against the same mid-gray homogeneous background, therefore whenever light and dark region are perceived a brightness illusion is generated.

In Figure 4A the inducers are aligned along a straight line alternating in gray shade and fall on opposite sides of an imaginary line. As in Figure 3A the CP across this line is invariant, consequently the aligned sides are predicted to bind. A second contour of invariant CP is assumed to conjoin the inducers outer perimeters (*axis* 2). Each column of inducers alternate with a column of opposite CP (see Figure caption).

The same configuration is reproduced in Figure 4B with a crucial variation in the inducers surface: they are split into two halves of approximately the same area and opposite gray shade with respect to the intermediate gray surround. This variation left the CP along the vertical a*xis* 1 unaltered, whereas the pathway along the external perimeter of the shapes periodically reverses the CP. Therefore, only one source of induction is active in Figure 4B, the vertical *axis* 1.

In Figure 3C even this source is inhibited since the inducers are reoriented so that to reverse the CP along both the two directions of contour completion.

If we compare the gray backgrounds, a striking first datum emerges: the background that in Figures 4A,B appears divided into dark and light regions is seen without differences in brightness in Figure 4C. This supports the hypothesis that the presence of *axes* of invariant CP coincides with the brightness alterations.

The role of the *axis* 1 of invariant CP is documented by the Figure 4B. Here the twin-shade inducers have inhibited the *axis* 2 completion while leaving the *axis* 1 unaltered. Note the alternation of dark and light vertical bands in the background. We can check that the direction of the change in polarity is predicted by the CP of the *axes*. Their locations are indicated by the bidirectional arrows and the CP polarity by the “+” and “−” at the opposite sides. For example, a background's illusory brighter region is embedded between −/+ and +/− pairs of *axes*.

The same axes (*axes* 1) complete in the top figure (the bidirectional arrow symbolize this correspondence) but in concomitance with the *axes* 2. The conditions now exist for the observer to make direct comparisons of the effects generated by two *axes* or by one only.

If we concentrate on the leftmost columns we can check that it appears to brighten in Figure 4B whereas a dark gray seems to fill it in Figure 4A. Nevertheless, the reversal of polarity induction is to be associated with a second evident illusion in Figure 4A: alternating vertical bands of different illumination. Between the leftmost two columns a rectangular beam of light is projected on a surface in which a surface bordered by a square waved contour is colored of dark gray.

This can be considered the “layered image” solution when a region of the background is passed through by induction signals of opposite CP. The solution emerges despite the violation of the geometrical constraints for the transparency to arise and confirms the role of the perceived transparency, or layering, in modulating brightness (Nakayama et al., 1990). The illusory alternation of light and dark regions separated by square-waved contours is striking, more vivid than the brightness alterations in Figure 4B where the depth stratification is absent. A further comparison of these induction effects will be carried out in a subsequent paragraph.

The first series of phenomenological observations lead to the following conclusions:

- The formation of an *axis* of invariant CP is a source of lightness/darkness induction signals;
- Opposite brightness signals give origin to a stratification of the image into layers.

An experiment has been designed to assess its consistency and relationship with important factors such as luminance contrast.

### Experiment

The test stimuli reproduce Figure 4B. This configuration allows observation of the induction effects of an invariant CP contours taken in isolation and that coincides with the line dividing the jagged shapes.

The target of the induction signals is the background of homogeneous gray: this simplifies the procedure followed so far for documenting the brightness induction effects that requires to discount the simultaneous brightness contrast (SBC) between background and the small target units drawn between the inducers (such as in the “snake illusion”).

Finally, use of the twin-shade inducers in this way allows neutralization of the sources of induction that in the “snake illusion” compete with the induction generated by an *axis* of invariant CP. Along the contour separating the background and the inducers we find alternating light and dark edges whose opposite SBC effects balance. A similar argument can be put forward to exclude effects of remote contrast and assimilation (2).

The main aim of the experiment was to document the induction effects under a variety of conditions, in order to eliminate the possibility of their being a product of the specific combination of aspects that occurs in Figures 2C, 4A.

The variations we introduced had the effect of altering the luminance ratios across the edges and their CP.

We drew *axes* of invariant CP with inducers of different gray shades so as to obtain different luminance ratios that allowed observation of how this affects the brightness. By rotating the same inducers we created *axes* of variable CP. The presence and magnitude of the illusion was probed by comparison of a figure in which the components formed *axes* of invariant CP against one with the same components but with rotation so that no invariant CP *axis* appeared (Figure 4C).

In accordance with previous findings on filling-in phenomena and edge integration that showed increase in induction with greater luminance ratios, we predicted stronger effects with higher-contrast inducers.

Only three different luminance ratios were introduced, sufficient to document a relation with strength of induction and gather information to support a subsequent fuller enquiry.

Our experiment tested two predictions:

- Alternating invariant CP *axes* (Figure 4B) will generate brightness/darkness induction phenomena; but when the inducers are reoriented so as to have alternating opposite CP (Figure 4C), no induction effect will appear;
- More intense induction effects are expected when the difference in luminance increases on the opposite sides of the invariant CP axes.

## Methods

The subjects viewed the two configurations (16 × 6.5 cm) simultaneously presented side by side on a CRT screen at a center-to-center distance of 17 cm, and matched the test configuration on the left (Figure 4B) with the comparison configuration on the right (Figure 4C). They were asked to perform a brightness-contrast task (Arend and Spehar, 1993; Rudd, 2010), in which they had to match brightness difference between the vertical bands on the right with the corresponding bands on the left. Viewing distance was 80 cm.

### Subjects

Twenty paid volunteers, unaware of the experiment's aims.

Stimulus display:

- Three types of inducers (~0.8 cm side) were drawn to give three levels of average luminance, and labeled HC, MC, and LC to indicate high, mean and low contrast degree; the inducers were split into halves having the following magnitudes (cd/m^2^): 0.2–54.2 (HC), 3.4–39.4 (MC), and 6.1–23.6 (LC).
- The same types of inducers were used for test and comparison stimuli. The axes of the inducer columns were 3 cm apart.
- The inducers could vary in orientation so as to obtain either invariant CP or variable CP axes.
- The background could be uniform gray (uniform background) or divided into alternating light and dark vertical bands (divided background). Two levels of average luminance were chosen.

Test stimuli:

- These factors were combined to give a total of 18 test stimuli, in three groups.
- Uniform background. The inducers were arranged as in Figure 4B to form an invariant CP *axis*. Five axes alternated in opposing CP. The three inducer types were combined with two background luminances (12.9 and 16.1 cd/m^2^), giving a total of six test stimuli.
- Divided background. The column axes coincided with boundaries of different background luminance (see Figure 4 caption). Three types of inducers combined with two pairs of background luminances (10.8 and 15.3 cd/m^2^, or 14.2 and 18.7 cd/m^2^), giving six stimuli.
- Control stimuli This set differed from the previous set (divided background) only in that some inducers were rotated so as to form axes of variable CP (Figure 4C). These configurations served to measure whether absence of axes of invariant CP allowed true judgment of brightness/darkness.

**Figure 5:**
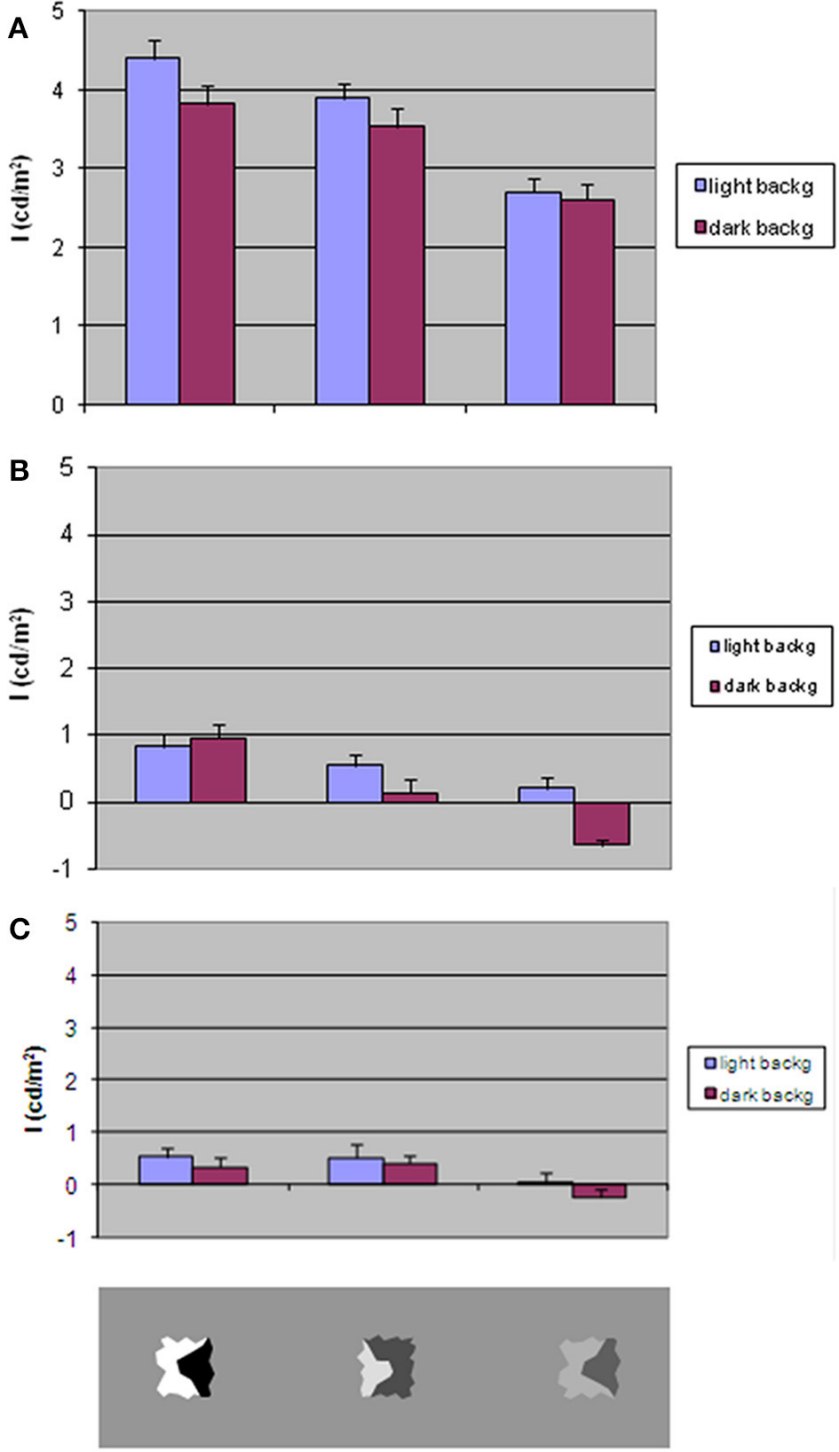
**The magnitude of the illusory brightness/darkness induction effect (index *I*) as a function of average background luminance and inducer contrast**. Bars indicate standard error. **(A)** Homogeneous surround. Test stimuli with invariant CP axes. **(B)** Divided background. Test stimuli with invariant CP axes. **(C)** Divided background. Test stimuli with axes of variable CP. Bottom gray rectangle: the three types of inducers. Left to right: HC (high contrast), mean contrast (MC), low contrast (LC).

Comparison stimuli:

The inducer orientation always formed variable CP axes (Figure 4C). For each of the 18 test stimuli, a series of comparison stimuli were generated by gradually differentiating the luminance of the vertical bands between the inducer axes. From an initial configuration with between-column background of common magnitude (12.9 or 16.1 cd/m^2^), successive stimuli were obtained by increasing the luminance (L between Figure 4C arrows) of one HSL point (Hue, Saturation, Luminance in the standard color dialog) and decreasing luminance (D between Figure 5 arrows) of the neighboring region of same magnitude. The mean variation was ~0.2 cd/m^2^. For each step the luminance of the darker and lighter bands was measured using a Minolta LS-110 photometer and the magnitude difference, L_p_-D_p_ (subscript p for photometric), calculated. The procedure was repeated 20 times to create a sequence of stimuli where luminance contrast between alternating bands gradually increased.

Care was taken to ensure that the reflectance edge separating the two background shades was hidden by the inducers.

The method of adjustment was used. The subjects sat at 1 m from the screen, and were asked to observe the configuration on the left (test), compare it with that on the right (comparison), and say whether they perceived differences in luminance between the two sides. They were then asked to adjust the difference in luminance between the vertical bands of the comparison, by clicking on one of two colored arrows at the bottom of the display to reproduce the same difference in luminance they had perceived in the test. They were instructed to neglect the characteristics of the irregular shapes and concentrate on the luminance of the background.

The 18 conditions were presented in random order four times: in two cases the initial comparison stimulus had homogeneous background, or bands of similar shade of gray; in the other two divided background was initially presented.

## Results

Each response indicated the luminance difference judged as maximally similar to that of the test stimuli. In order to obtain a measure of the difference between estimated and real luminance differences, they were subtracted. The simple formula *I* = (L_p_-D_p_) − (L_s_-D_s_) [here *I* is magnitude of brightness illusion; L and D indicate lighter and darker luminances; subscript s is subjective value] measures how the perceived difference diverges from the real one; *I* was calculated for each trial and averaged across the four repetitions of the same condition.

Results obtained with homogeneous and divided backgrounds underwent separate ANOVA tests on mean values of *I*.

### Contrast polarity effects on homogeneous background

Figure 5A shows *I*-values, obtained with light and dark background, for the three levels of inducer contrast.

ANOVA revealed stronger effects with lighter background [*F*_(1, 19)_ = 7.33; *p* < 0.05] and with high inducer contrast levels [*F*_(2, 38)_ = 63.68; *p* < 0.001].

### Contrast polarity effects on divided background

Figure 5B shows the mean *I*-values as a function of average background luminance and inducer types in the condition where test stimuli have invariant CP *axes*. Figure 5C shows the mean *I*-values in relation to the same factors when the test stimuli have variable CP. The patterns of results are clearly very similar, as confirmed by the statistical test, and a mean magnitude illusion negligible with respect to the homogeneous background condition.

A 2 × 2 × 3 ANOVA was calculated on the mean values in the following conditions: invariant vs. variable CP of axes, light vs. dark background average luminance, inducer types. The CP along the axes yielded no significant statistical difference in the two cases in which it varied or persisted. Background luminance was shown to also affect the judgments [*F*_(1, 19)_ = 12.05; *p* < 0.005] and inducer type [*F*_(2, 38)_ = 20.23; *p* < 0.001]. Inducer type affects the results differently in the two levels of background mean luminance [*F*_(2, 38)_ = 4.22; *p* < 0.05] and in the conditions of CP [*F*_(2, 38)_ = 5.83; *p* < 0.01].

Judgments then diverge only minimally from the true one when the background contains different gray shades. It is nonetheless important to note that some of these small values have statistical significance. A Student's *t*-test revealed that HC inducers produce a brightness induction of ~0.8 cd/m^2^ with invariant CP *axes*, but an induction in the opposite direction arises with LC inducers drawn on a darker background. HC inducers generate a brightness induction also with variable CP *axes*.

The main results can be summarized as follows. An invariant CP *axis* generates a brightness/darkness induction on opposite sides congruent with its CP. The strength of the effect varies in direct relation to the contrast magnitude across the *axis*. This effect is not observed when the background has different luminance from the sides of the *axis*.

Since this condition coincides with larger luminance differences across the edges, the prediction that induction effects vary directly in relation to this parameter needs to be revised. Possible approaches for investigating this point are discussed in the Conclusions.

#### Combined induction effects of ICP axes on the occurrence of brightness illusions

The second hypothesis put forward here asserts that overlapping induction signals of opposite CP are at the origin of the decomposition into layers. Figures 2C, 4A have been described as supporting the hypothesis, Figure 1 also “argues” in favor. Note that the trunk and legs of the statue, or rather the area where the conical filter and statue overlap, are traversed by induction signals of the opposite sign. This interference should be considered a crucial event similar to problem-solving (Rock, 1983), which requires a solution. In fact, two induction signals conflicting in one same region is equivalent to saying that two surface completion processes compete in the same portions of a two-dimensional space. A good solution to the conflict would be to separate the two streams, assigning them different layers. This is simply the solution the visual system adopts. The retinal image is decomposed into a reflectance and a shading component, both with linear contour and brightness/lightness. The beam of shadow appears as a filter and the statue shape as a light silhouette on the background. The two opposite sign induction streams, once separated into two layers, lead to the perception of a dim filter (or shadow) and a light gray surface beneath (the “winged victory”).

The layering is not the result of a computation of local cues such as the X-junctions but the solution of conflicting-filling-in processes. To test this hypothesis we have prepared some patterns that allow observation of the target brightness variation when the induction does or does not interfere.

**Figure 6:**
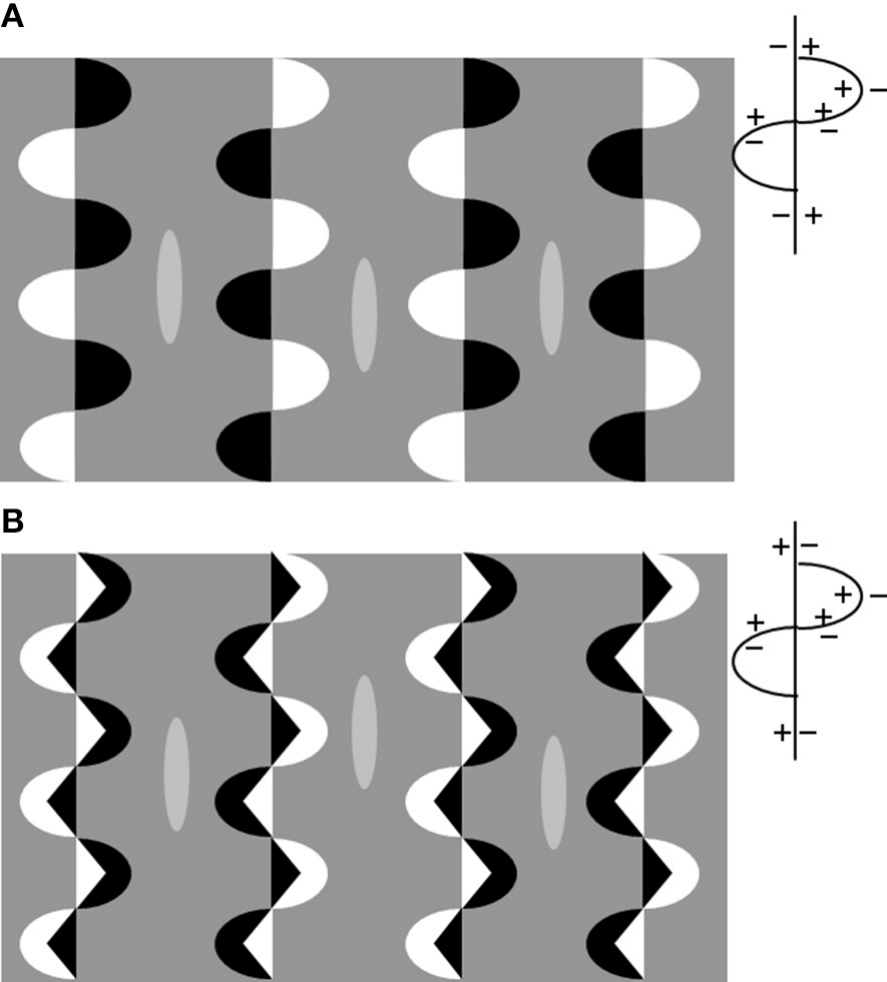
**(A)** The iso-contrast version of the “snake illusion”: the two axes (straight and wavy) have opposite CP as illustrated in the outline patterns on the right. The background between the columns of inducers is traversed by induction signals of the opposite sign. Three elongated ellipsoids drawn in the central portion of the columns serve as targets. They are identical in gray shade but the central one looks darker. **(B)** Same as in **(A)** but with smaller inducers inside the semi-ellipses that generate vertical axes of the opposite sign with respect to **(A)** as illustrated in the outline on the right. The background is traversed by induction signals of the same sign. In this case the interlaced axes sum their effects. Note the target brightness; the differences are negligible with respect to that observed in **(A)**.

In Figure 6A we have reproduced a variation of the “snake-illusion” in which the background is a homogeneous gray. The axes generate inductions of opposite sign on the background as illustrated in Figure 3. Figure 6B has been obtained by inverting the CP of the vertical *axis* so as to give two interlacing edges of the same CP orientation. With this combination of *axes*, the background between two columns of inducers is traversed by two streams of induction signals of the same sign (see outline pattern in Figure 6). We expect no layering to occur. If we compare the brightness alteration of the three targets (the ellipses have an identical gray shade) we observe a marked difference in the upper configuration but a negligible effect in the lower configuration. Note that in this latter case the background is viewed as split into vertical light and dim bands, in agreement with predictions that can be made considering the CP of the vertical *axes*. It is interesting to note that the brightness alterations are more vivid in the layered image (Figure 6A).

To conclude, one invariant CP *axis* induces brightness/darkness on the background. If a second set of *axes* is introduced that propagates induction of the same sign on the background, the effects persists. The targets (in the form of small surfaces) on these portions of the background seem insensitive to the induction signals.

Their brightness is found to alter when the two sets of *axes* propagate on the same portion of the background opposite induction signals. In this case, when the image splits into a reflectance and an illumination layer, the small target-figures (the diamonds in “snake illusion,” the ellipsoids in Figure 6) are perceptually placed at the reflectance layer and darken or brighten in accordance with the induction signals at this level.

## Conclusions

The findings reported here confirm the hypothesis that the formation of contours from edges of the same CP generates the brightness/darkness induction effects. These effects combine in different patterns of interference that have different phenomenological outcomes. For these effects—both binding and induction—to arise it not necessary that four surfaces converge at the same point as seen in the image of an X-junction. The edges can be non-collinear and their endings can be separated by a gap; in fact if the distance and the deviation of colinearity are within a short range (Roncato and Casco, 2003, 2006) the effects are clearly visible. This explains why we perceive induction effects even with configurations where the X-junctions are not replicated (Bressan, 2001).

Furthermore, on the same area they may propagate induction signals from different combination of *axes* of the invariant CP, leading to different brightness effects. When the induction signals are of the opposite sign, it is likely that the visual system uses them as cues to decompose the image into layers.

Some open questions remain, which should form the basis of a subsequent study.

The first question addresses the nature of the induction effects generated by the invariant CP *axes*. The *axes* we produced in Figures 2–4 are only partially adjacent to the induced surfaces (background) because the “contact edges” alternate with areas of no contact. The conclusion is that the induction signals do not originate along the edges but from a complex unit that integrates shorter edges and becomes a source propagating into the adjacent and non-adjacent surfaces. Rudd's model (Rudd, 2010, 2013) predicting spreading of induction signals beyond edges and strategies of integration offers an account of the findings reported here, but the stimulus conditions of his research differ from those created here so that a complete test cannot be made.

The brightness perception is altered at a negligible magnitude or not at all when the luminance of the regions embedded between the invariant CP *axes* are different (Figure 5B).

This an unexpected finding.

It could be one of two things. Il might be that *axis* 1 does not act as a source of dark/light signals emission. Or it might be that these signals do not combine or sum to the signals (the edge induction signals from the real edges, for example) that allow the observers to give correct judgments when the *axes* 1 do not complete (Figure 5C). In other words the induction signals propagating from the illusory contour (*axis* 1) cause visible effects on a background of homogeneous luminance but the same effects are not detected when concomitant dark/light signals spread from real high contrasted contours. Previous research on contrast matching does not report findings that can be useful to the understanding of the “anomaly” in Figure 5B findings.

**Figure 7:**
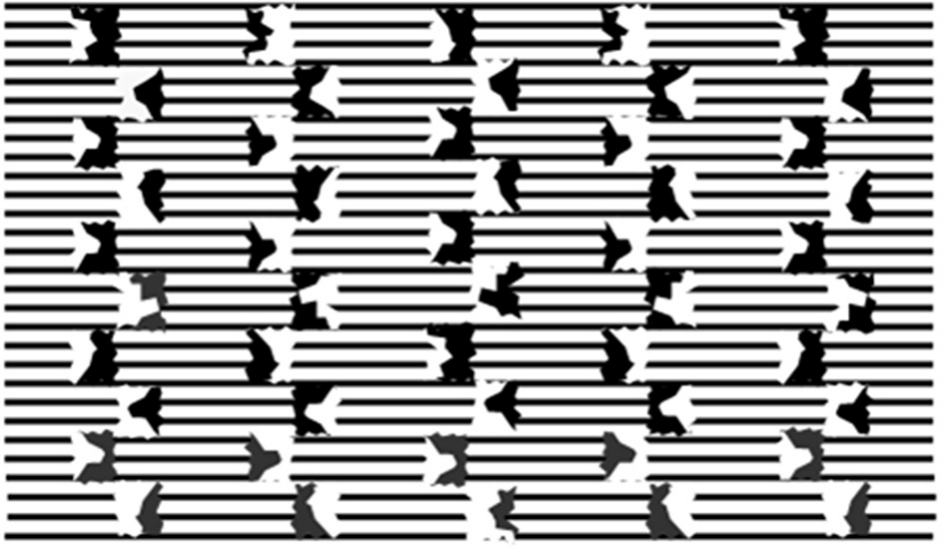
**Split inducers as in Figure 4A against a background of parallel black lines and white interspaces**. The impression that the columns embedded between inducers are of different brightness is illusory.

The brightness/darkness induction signals from the invariant CP manifest a further property as illustrated in Figure 7. Here the inducer columns, similar to those of Figure 4B are drawn against a textured background made up of parallel black lines.

Light and dark columns appear to alternate at the sides of the imaginary axes dividing the inducers. At close inspection, the origin of this impression is mysterious since any change in line shades and thickness is absent all along their extension. The same is valid for the white interspaces between them. To conclude, in Figure 7 neither physical nor phenomenal alterations are present that justify the appearance of light and dark columns. It is likely that the background is processed as a textural surface, that is a figural organization that behaves like regions of homogeneous color when gaps are filled by the filling-in phenomena (Spillmann and De Weerd, 2003) or when neon spreading occurs (Watanabe and Cavanagh, 1991). The same interpolation processes are likely to act in Figure 7, too. In this case they act as a source of lightness/darkness induction. Since there are no surfaces to be darkened or lightened the outcome is phenomenological ambiguous; we are uncertain about the nature of the background differences: whether a difference in illumination, in texture density, line thickness, and so on.

Illusory phenomena such as the one shown here demonstrate that the phenomenological analysis and methods are still very relevant and suitable to unveil aspects useful to deepen our understanding of perceptual organization.

1. The term “brightness” is used to mean “perceived luminance,” i.e., the light we perceive in a surface, “lightness” stands for “perceived reflectance” or the pigment of a surface. The distinction is useful when the illumination component is clearly visible in the stimulus. Some researchers indicate which dimension has been measured, others uses only one of the two terms to refer to the perceived gray of an achromatic surface. I choose the term “brightness” throughout the text because it is not possible to establish, for the main effect under investigation (see Figure 4B), the belonging to one of the two classes of misperception phenomena: the “perceived luminance” or the “perceived reflectance” alteration.
2. Furthermore, the use of the twin-shade shapes allows us to eliminate the alteration of brightness as a consequence of “atmosphere effects” (Adelson, 2000) or “anchoring effects” (Bressan, 2006).

### Conflict of interest statement

The author declares that the research was conducted in the absence of any commercial or financial relationships that could be construed as a potential conflict of interest.
